# Costs of Severe Maternal Morbidity in U.S. Commercially Insured and Medicaid Populations: An Updated Analysis

**DOI:** 10.1089/whr.2021.0026

**Published:** 2021-09-27

**Authors:** Christopher M. Black, Kimberly K. Vesco, Vinay Mehta, Pamela Ohman-Strickland, Kitaw Demissie, Dona Schneider

**Affiliations:** ^1^ School of Public Health, Rutgers University, Piscataway, New Jersey, USA.; ^2^ Merck & Co., Inc., Center for Observational and Real-World Evidence (CORE), Kenilworth, New Jersey, USA.; ^3^ Kaiser Permanente Center for Health Research, Portland, Oregon, USA.; ^4^ School of Public Health, SUNY Downstate Health Sciences University, Brooklyn, New York, USA.; ^5^ Edward J. Bloustein School of Planning and Public Policy, Rutgers University, New Brunswick, New Jersey, USA.

**Keywords:** comorbidity, health care costs, hospitalization, insurance claim review, pregnancy complications

## Abstract

***Background:*** The most common reason for hospitalization in the United States is childbirth. The costs of childbirth are substantial.

***Materials and Methods:*** This was a retrospective cohort study of hospital deliveries identified in the MarketScan^®^ Commercial and Medicaid health insurance claim databases. Women with an inpatient birth in the calendar year 2016 were included. Severe maternal morbidity (SMM) was identified using the Centers for Disease Control and Prevention algorithm of 21 International Classification of Diseases-10 codes. Mean costs and cost ratios for women with and without SMM were reported. Generalized linear models were used to analyze demographic and clinical variables influencing delivery costs.

***Results:*** We identified 1,486 women in the Commercial population, who had a birth in 2016 and met the criteria for SMM. The total mean per-patient costs of care for women with and without SMM were $50,212 and $23,795, respectively. In the Medicaid population there were 29,763 births, of which 342 met the criteria for SMM. The total mean per-patient costs of care for women with and without SMM were $26,513 and $9,652, respectively. A multifetal gestation, a cesarean delivery, maternal age, and pregnancy-related complications were independently predictive of increased delivery costs in both Commercial and Medicaid populations.

***Conclusions:*** The occurrence of SMM was associated with an increase in maternity-related costs of 111% in the Commercial and 175% in the Medicaid population. Some of the factors associated with increased delivery hospitalization costs could be treated or avoided.

## Introduction

The most common reason for hospitalization in the United States is childbirth.^1^ The costs of childbirth are substantial and are increased by a cesarean delivery, multifetal pregnancy, preexisting medical conditions, and complications of pregnancy.^2–6^ A cesarean delivery, multifetal gestation, and maternal comorbidities are also risk factors for severe maternal morbidity (SMM),^7–12^ which refers to scenarios in which women experience one or more life-threatening complications during labor or delivery.^13^ The incidence of SMM in the United States has been estimated as 144 per 10,000 delivery hospitalizations.^13^ In studies based on hospital discharge records, mean delivery costs (not including prenatal and postdelivery costs) associated with SMM were estimated to be about twice those of deliveries without SMM.^14,15^

We previously described the use of insurance claims data to determine prenatal, delivery, and postpartum costs in commercially insured and Medicaid patients in 2013 and found that mean costs were greater with deliveries with SMM compared with deliveries without SMM.^16^ However, in these previously reported studies, SMM was identified using the Centers for Disease Control and Prevention (CDC) list of 25 indicators, based on the International Classification of Diseases, 9th edition (ICD-9) codes.^14–16^ In 2017 the CDC algorithm for SMM was revised from 25 indicators to include a list of 21 indicators based on ICD-10 codes.^13^ The incidence of SMM in nationwide samples of commercially and Medicaid-insured women using the revised CDC algorithm for SMM has been reported recently and found the incidence to be 111.4 in the Commercial and 109.6 per 10,000 deliveries in the Medicaid population.^17^ The objective of the present analysis was to determine the costs of prenatal care, hospital delivery, and postdelivery care in commercially and Medicaid-insured women with and without SMM, using the revised CDC algorithm for SMM, and to determine the relationship between delivery costs and various risk factors.

## Materials and Methods

### Study overview

This was a retrospective cohort study of SMM in beneficiaries enrolled in the MarketScan^®^ Commercial Claims and Encounters (“Commercial”) and Medicaid databases. The Commercial database includes paid medical and prescription drug claims for several million individuals annually from ∼300 self-insured U.S. employers and 25 health plans, while the Medicaid database contains the pooled health care experience of Medicaid enrollees from 11 states.^17,18^ Due to privacy agreements between the Medicaid state agencies and the agency that manages the coordination of benefits, the states included are not publicly identifiable. The MarketScan data were deidentified, so that Institutional Review Board approval was not required.

### Study sample

The study population included all women with an inpatient birth in 2016, identified by ICD-10 diagnostic and procedural, Current Procedural Terminology, and Diagnosis-Related Group codes for a delivery (Supplementary Table S1).^19^ In the primary analysis, continuous enrollment (without gaps) was required during the 9 months before and 30 days following the delivery.

SMM was defined by the occurrence during delivery hospitalization of one or more of the 21 potentially life-threatening conditions or complications identified by the diagnostic and procedural ICD-10 codes identified by the CDC (Supplementary Table S2).^13^ Preexisting comorbidities and obstetric-related complications were identified from the literature and the corresponding ICD-10 codes identified (Supplementary Table S3).^20–22^ Patient characteristics included in the study were maternal age (in 5-year age groups), gestation type (singleton or multifetal), delivery method (vaginal or cesarean), geographic region (Commercial population only), and race/ethnicity (Medicaid population only). Geographic regions included the four divisions defined by the U.S. Census: Northeast, Midwest, South, and West. Race/ethnicity was categorized as White non-Hispanic, Black non-Hispanic, Hispanic, and other.

### Cost analysis

Costs are presented in 2016 U.S. dollars. All-cause costs incurred, whether obstetrics related or not, were classified into one of three time periods: the prenatal period (9 months before delivery hospitalization admission), delivery period (costs during hospitalization), and postdelivery period (30 days following delivery hospitalization discharge). Total costs were determined for all services, using the amounts paid by insurers plus out-of-pocket and third-party payments. Cost data were calculated as mean, standard deviation. Costs were also calculated as median and interquartile range. Costs were calculated by SMM status, delivery method, gestation type, maternal age category, region in the Commercial population, and race in the Medicaid population. Comparisons of mean costs between women with and without SMM were made using the *t*-test. Generalized linear models were used to analyze the relationship between demographic and clinical variables and delivery costs, adjusting for preexisting conditions and pregnancy-related complications. Generalized linear model methodology is an appropriate technique for analyzing skewed cost data.^23^ The models used a log link and gamma error distribution function that conforms to the non-normal cost distributions typical of health care costs. Results are presented as cost ratios and mean costs with 95% confidence intervals (CIs). For the Medicaid population, a sensitivity analysis was conducted for delivery costs, in which the continuous enrollment criterion was removed. This sensitivity analysis was completed to account for potential selection bias in the Medicaid population, which was observed to have a fragmented enrollment history. All analyses were conducted using SAS Version 9.4.31.

## Results

### Commercial population

**FIG. 1.**
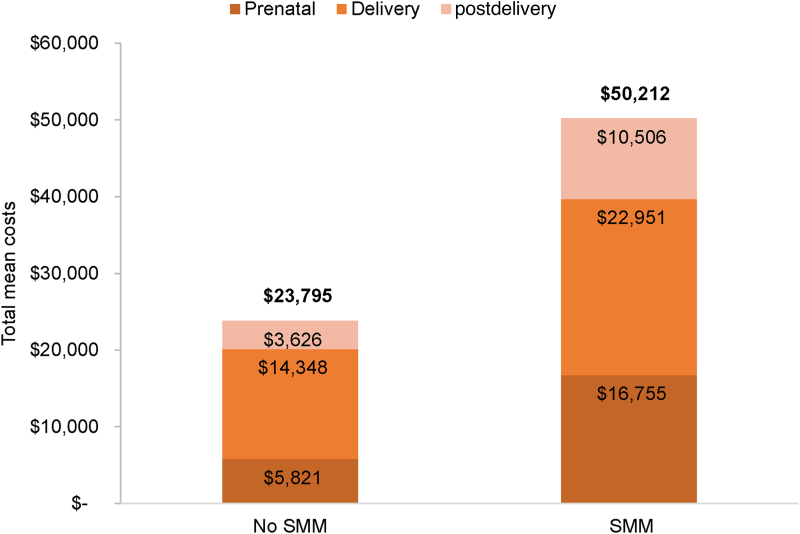
Total mean cost across time periods by SMM status in the Commercial population. Total costs are in bold on top of the bar for women without SMM. *p* < 0.001 for comparisons of prenatal, delivery, and postdelivery costs in women with and without SMM. SMM, severe maternal morbidity.

**FIG. 2.**
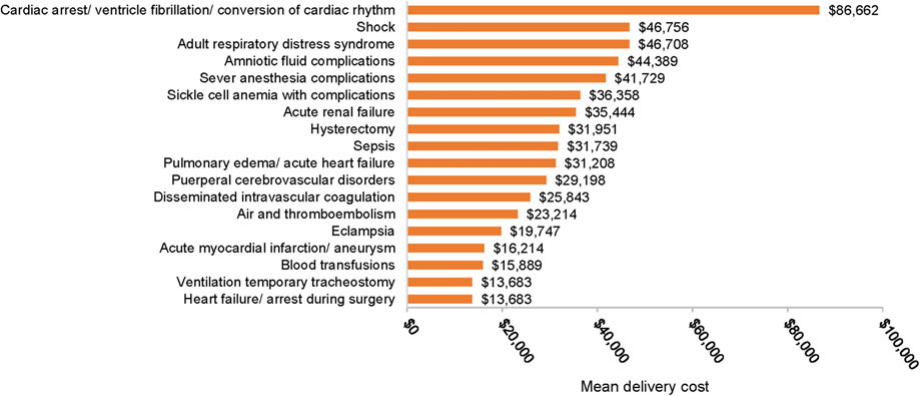
Mean delivery cost by SMM indicator in the Commercial population.

A total of 130,297 delivery hospitalizations met the study inclusion criteria, of which 1,486 deliveries were classified with SMM, for a rate of 114 per 10,000 deliveries. The total mean per-patient costs of care for women with and without SMM were $50,212 and $23,795, respectively (Fig. 1). Prenatal, delivery, and postdelivery costs were all significantly higher at *p* < 0.001 for women with SMM versus women without SMM (Fig. 1). Delivery costs comprised the greatest proportion of total costs, which is 45.7% of total costs in women with SMM and 60.3% of women without SMM. Among women with SMM, prenatal costs comprised 33.4% of total costs, whereas postdelivery costs comprised 20.9% of costs. Similarly, among women without SMM, prenatal, and postdelivery costs comprised 24.5% and 15.2% of total costs, respectively. The individual SMM indicator associated with the greatest delivery cost ($86,662) was cardiac arrest/ventricular fibrillation/conversion of cardiac rhythm (Fig. 2).

In the generalized linear model analysis, SMM increased delivery costs by 20% and was the strongest predictor of delivery costs, with an adjusted mean (95% CI) cost of $22,672 ($21,277–$24,154; Table 1). Relative to age 31–35 years, the 19–24 and 25–30 years age categories were predictive of lower delivery costs, and age categories >35 years were significantly associated with increased delivery costs. In addition, delivery method, gestation type, Northeast geographic region, and presence of any obstetric complication or preexisting comorbidity were independently predictive of increased delivery costs (Table 1).

**Table 1.** Predictors of Adjusted Delivery Cost in the Commercial Population

	Adjusted cost ratio (95% CI)	Adjusted mean cost (95% CI)
Intercept	—	$15,607^a^
SMM (referent: no SMM)	1.20 (1.17–1.24)^b^	$22,672 ($21,277–$24,154)
Delivery (referent: vaginal)		
Cesarean	1.17 (1.17–1.18)^b^	$22,103 ($21,220–$23,024)
Maternal age (referent: 31–35)		
14–18	0.94 (0.85–1.05)	$17,781 ($15,430–$20,490)
19–24	0.94 (0.92–0.96)^b^	$17,692 ($16,726–$18,715)
25–30	0.96 (0.94–0.97)^b^	$18,041 ($17,177–$18,949)
36–40	1.02 (1.01–1.04)^b^	$19,287 ($18,339–$20,282)
41–45	1.08 (1.04–1.11)^b^	$20,243 ($18,993–$21,575)
>45	1.09 (1.00–1.20)^b^	$20,590 ($18,151–$23,356)
Gestation status (referent: singleton)		
Multifetal	1.09 (1.07–1.11)^b^	$20,422 ($19,372–$21,528)
Region (referent: West)		
Midwest	0.86 (0.85–0.87)^b^	$16,242 ($15,522–$16,995)
Northeast	1.22 (1.20–1.23)^b^	$22,929 ($21,890–$24,017)
South	0.85 (0.85–0.86)^b^	$16,045 ($15,366–$16,756)
Pregnancy-related complication (referent: no presence of complications)	1.04 (1.04–1.05)^b^	$19,633 ($18,843–$20,457)

^a^ The intercept represents the adjusted mean cost estimate for a woman with no SMM, vaginal delivery, age 31–35, singleton, West, and no presence of complications.

^b^ Multivariate regression analysis statistically significant at *p* < 0.05.

CI, confidence interval; SMM, severe maternal morbidity.

### Medicaid population

**FIG. 3.**
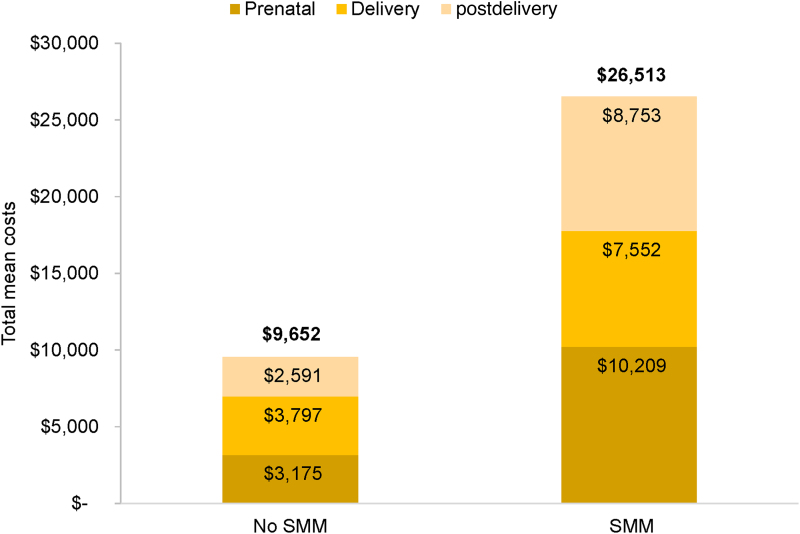
Total mean cost across time periods by SMM status in the Medicaid population. Total costs are in bold on top of the bar for women without SMM. *p* < 0.001 for comparisons of prenatal, delivery, and postdelivery costs in women with and without SMM.

**FIG. 4.**
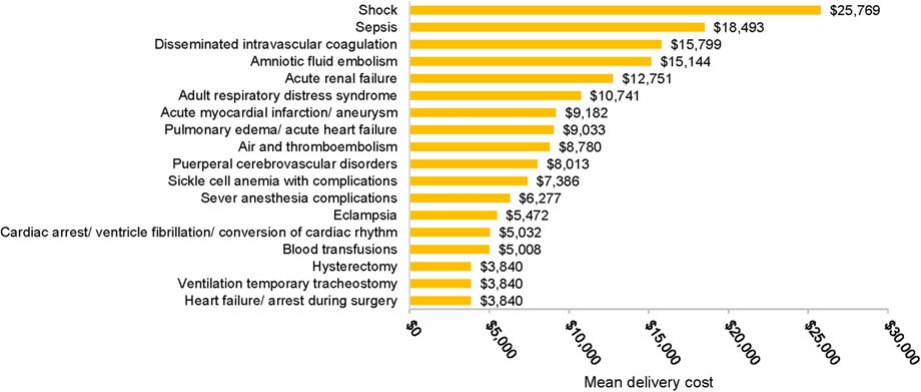
Mean delivery cost by SMM indicator in the Medicaid population.

A total of 29,763 delivery hospitalizations met the study inclusion criteria, of which 342 deliveries were classified with SMM, for a rate of 115 per 10,000 deliveries. The total mean per-patient costs of care for women with SMM ($26,513) were 177% higher than for women without SMM ($9,652; Fig. 3). Costs of prenatal, delivery, and postdelivery costs were all higher for women with than without SMM (*p* < 0.001 for all comparisons). Delivery costs were 39.3% of total costs for women without SMM, and 28.5% of total costs for women with SMM. The SMM indicator associated with the greatest delivery cost was shock ($25,769; Fig. 4).

In the generalized linear model analysis, the adjusted delivery cost was 31% greater for women with than for women without SMM (Table 2). Adjusted mean delivery costs were significantly higher for women ages 31–35 compared with women 19–30 years of age, for Black non-Hispanic versus White non-Hispanic women, for a cesarean versus vaginal delivery, a multifetal versus singleton gestation, and with the presence of any obstetric complication or preexisting comorbidity (Table 2).

**Table 2.** Predictors of Adjusted Delivery Cost in the Medicaid Population

	Adjusted cost ratio (95% CI)	Adjusted mean cost (95% CI)
Intercept	—	$5,932^a^
SMM (referent: no SMM)	1.31 (1.23–1.40)^b^	$7,796 ($6,772–$8,974)
Delivery (referent: vaginal)		
Cesarean	1.16 (1.15–1.18)^b^	$6,908 ($6,300–$7,575)
Maternal age (referent: 31–35)		
14–18	0.96 (0.89–1.03)	$5,674 ($4,886–$6,589)
19–24	0.96 (0.92–0.99)^b^	$5,667 ($5,051–$6,359)
25–30	0.96 (0.93–0.99)^b^	$5,706 ($5,085–$6,402)
36–40	1.03 (0.97–1.09)	$6,122 ($5,337–$7,022)
41–45	1.08 (0.96–1.21)	$6,395 ($5,262–$7,773)
>45	1.03 (0.70–1.53)	$6,138 ($3,831–$9,835)
Gestation status (referent: singleton)		
Multifetal	1.10 (1.06–1.15)^b^	$6,530 ($5,791–$7,363)
Race/ethnicity (referent: Black)		
Hispanic	1.00 (0.95–1.05)	$5,943 ($5,224–$6,761)
Other	1.03 (0.97–1.09)	$6,105 ($5,323–$7,000)
White	0.97 (0.94–0.99)^b^	$5,742 ($5,172–$6,373)
Pregnancy-related complication (referent: no presence of complication)	1.03 (1.01–1.05)^b^	$6,101 ($5,551–$6,706)

^a^ The intercept represents the adjusted mean cost estimate for a woman with no SMM, vaginal delivery, age 31–35, singleton, Black, and no presence of complications.

^b^ Multivariate regression analysis statistically significant at *p* < 0.05.

In the sensitivity analysis, conducted without the continuous enrollment criterion, there were 219,670 delivery hospitalizations in the Medicaid population, with a similar rate of SMM (110 per 10,000 deliveries) as in the primary analysis (115 per 10,000 deliveries). Total mean delivery costs with or without SMM in the sensitivity analysis were twice those in the primary analysis (Table 3). Nevertheless, the cost ratios between women with SMM and no SMM were similar (1.99 and 1.98 in the primary and sensitivity analyses, respectively), for a cost ratio difference of −1.0% (Table 3). The greatest differences in mean cost ratios in the sensitivity versus the primary analysis were observed in the categories of race/ethnicity and gestation type: −1.16 for “other” race/ethnicity, and −1.44 for a singleton gestation.

**Table 3.** Mean Delivery Costs in the Primary and Sensitivity Medicaid Analyses

	Primary analysis			Sensitivity analysis			Cost ratio difference^b^
	No SMM	SMM	Cost ratio^a^	No SMM	SMM	Cost ratio^a^	
	Mean ± SD	Mean ± SD		Mean ± SD	Mean ± SD		
Total	$3,797 ± 1,742	$7,552 ± 12,197	1.99	$7,677 ± 8,849	$15,210 ± 30,229	1.98	−0.01
Age, years							
14–18	$3,681 ± 2,446	$6,319 ± 2,384	1.72	$6,597 ± 6,215	$9,455 ± 8,818	1.43	−0.29
19–24	$3,677 ± 1,398	$7,791 ± 17,727	2.12	$7,850 ± 9,068	$14,216 ± 26,424	1.81	−0.31
25–30	$3,774 ± 1,517	$6,742 ± 6,012	1.79	$7,746 ± 8,967	$15,035 ± 27,729	1.94	0.15
31–35	$3,971 ± 2,277	$7,626 ± 7,395	1.92	$7,421 ± 8,497	$16,757 ± 35,297	2.26	0.34
36–40	$4,167 ± 2,128	$8,907 ± 12,938	2.14	$7,455 ± 8,546	$16,879 ± 40,777	2.26	0.12
41–45	$4,314 ± 3,524	$9,981 ± 13,532	2.31	$7,562 ± 8,958	$13,723 ± 16,636	1.81	−0.50
>45	$4,303 ± 1,097	$6,597 ± 6,215	1.53	$7,413 ± 9,180	$11,656 ± 5,096	1.57	0.04
Delivery method							
Cesarean	$4,787 ± 2,468	$8,592 ± 8,676	1.79	$10,013 ± 11,467	$16,844 ± 28,782	1.68	−0.11
Vaginal	$3,430 ± 1,186	$6,524 ± 14,837	1.90	$6,702 ± 7,273	$13,302 ± 31,743	1.98	0.08
Gestation type							
Singleton	$4,911 ± 5,361	$13,648 ± 35,699	2.78	$10,658 ± 13,341	$14,259 ± 23,045	1.34	−1.44
Multifetal	$3,770 ± 1,463	$6,649 ± 6,950	1.76	$7,676 ± 8,721	$14,974 ± 30,659	1.95	0.19
Race/ethnicity							
Black	$3,878 ± 1,653	$7,676 ± 15,202	1.98	$15,762 ± 14,467	$41,600 ± 60,365	2.64	0.66
Hispanic	$3,791 ± 2,071	$5,422 ± 3,048	1.43	$8,616 ± 9,565	$14,636 ± 28,981	1.70	0.27
Other	$3,973 ± 2,607	$11,548 ± 13,937	2.91	$5,674 ± 6,003	$9,929 ± 12,761	1.75	−1.16
White	$3,676 ± 1,594	$7,186 ± 8,073	1.96	$7,887 ± 9,601	$16,742 ± 24,568	2.12	0.16

^a^ Unadjusted cost ratio.

^b^ The cost ratio difference represents the difference between the sensitivity and primary analysis cost ratios.

SD, standard deviation.

## Discussion

In this analysis, the occurrence of SMM during delivery hospitalization was associated with an increase in total mean health care costs of 111% and 175% in the Commercial and Medicaid populations, respectively. SMM was more strongly predictive of increased delivery hospitalization costs than a cesarean delivery, maternal age, or multifetal gestation. Pregnancy-related complications were independently associated with increased delivery costs in both the Commercial and Medicaid populations. Furthermore, racial and ethnic disparities were observed in the Medicaid population, with elevated costs associated with SMM among Black non-Hispanic women compared with White non-Hispanic and Hispanic women.

Prenatal, delivery, and postpartum costs were all considerably higher in the Commercial than in the Medicaid population in both women with or without SMM. Commercial insurance is known to reimburse for services at higher payment rates compared with government sponsored insurance.^24^ Comparing the present 2016 cost analysis with the previous 2013 analysis,^16^ total pregnancy-related costs with or without SMM were considerably higher in 2016 in both the Commercial and Medicaid populations: total costs in the Commercial and Medicaid populations were 40%–60% higher without SMM and 150%–260% higher with SMM. Delivery costs with SMM were 48% higher in 2016 than in 2013 in the Commercial population, but similar (4% higher) in 2016 compared with the 2013 Medicaid populations—which suggests that the change in SMM coding algorithm did not itself increase costs.

Two earlier studies reported delivery hospitalization costs associated with SMM, using, respectively, discharge data from the 2011 National Inpatient Sample (NIS) and a dataset of all delivery hospitalizations in New York City (NYC) from 2008 to 2012.^14,15^ Direct comparison of these delivery cost estimates with estimates based on reimbursements in separate Commercial and Medicaid populations is difficult, given the different population structures and cost data sources. The earlier analyses estimated the costs expended by the hospital but excluded physician expenses. In the present study and in the previous 2013 analysis,^16^ populations of employer-sponsored insurance beneficiaries and Medicaid beneficiaries were analyzed separately. In contrast, the NIS and NYC studies analyzed mixed populations of commercially insured and Medicaid patients (the former 50% Commercial and 44% Medicaid patients and the latter 38% Commercial and 58% Medicaid patients).^14,15^ In addition, the earlier studies used an algorithm for identifying SMM based on ICD-9 codes, whereas the present study was based on ICD-10 codes.^14–16^

The accuracy of SMM algorithms based on either ICD-9 or ICD-10 codes depends on the correct assignment of codes in hospital discharge records. In a study set in the period 2001–2011, in which the ICD-9 codes for SMM indicators were checked against medical records, the codes were correctly assigned in 82%–86% of instances, although this varied substantially by code.^25^ Furthermore, the sensitivity and predictive value of the ICD-10 version of the CDC algorithm for SMM have not been determined.

Removing the requirement for continuous enrollment increased the number of delivery hospitalizations in the sensitivity analysis of the Medicaid population. The requirement for continuous enrollment especially impacts the Medicaid population due to the fragmented patterns of enrollment. Delivery costs in the sensitivity analysis were greater overall and across all demographic and delivery characteristics, although the SMM cost ratios were similar. The increased costs could be attributed to the fragmentation of care, in that complications may not be identified or treated in a timely manner. Noted differences in the demographic and clinical profile of the women in the sensitivity analysis could explain the increased costs, but further research will be necessary to understand the specific cost drivers.

The continuous enrollment criterion may have reduced the ability to infer toward the larger base population, who may not have continuous enrollment for different reasons, such as job loss or changing employment status. The differences in absolute costs between the primary and sensitivity analyses of the Medicaid population could indicate potential selection bias. Since delivery hospitalization costs were restricted to those incurred at any time during 2016, prenatal and postdelivery costs were likely underestimated. Data for a full 9-month prenatal period were available only for deliveries in the last 3 months of 2016. Similarly, the costs of a 30-day postdelivery period were available only for deliveries in the first 11 months of 2016. The Medicaid database represented only births from 11 states in 2016 and it is not possible to generalize these results to a national population. Furthermore, due to privacy agreements between the Medicaid state agencies and Truven, the identities of the states included are not publicly available. Patients' racial/ethnic identities were not available in the Commercial population. The explanation of racial/ethnic disparities in maternal morbidity in the Medicaid population is unclear, but they do not appear to be explained by differences in patient characteristics or by delivery hospital.^26^ Analysis of cost data is problematic, because the data tend to be non-negative and positively skewed, with heavy tails. In this data set, as is typical, median costs were lower than means in both the Commercial and Medicaid populations (Supplementary Tables S4 and S5). Nevertheless, the differences in median costs for women with and without SMM mirrored those for mean costs.

The data in this study indicate that there may be financial incentives for initiatives to reduce maternal morbidity. In the predelivery period, many of the pregnancy-related complications (gestational hypertension, preeclampsia, obstetric infection, *etc*.) associated with higher costs are preventable. There is evidence supporting the implementation of quality improvement programs designed to reduce maternal morbidity.^27^ Collections of best practices, or care bundles, have been developed to address obstetric hemorrhage and severe hypertension in particular.^27–29^ In the postdelivery period, the American College of Obstetricians and Gynecologists has recommended that postpartum care should become an ongoing process, with services tailored to each woman's individual needs.^30,31^ In addition, the fragmentation of care in the Medicaid population could be addressed by modifying eligibility and enrollment criteria. Our data suggest that the costs of these initiatives could be offset by reductions in costs associated with SMM.

## Conclusion

In conclusion, women experiencing SMM incurred significantly greater health care costs during the prenatal, delivery hospitalization, and 30-day postdelivery periods compared with women without SMM. Several demographic factors, preexisting comorbidities, and obstetric complications were associated with increased delivery hospitalization costs.

## Supplementary Material

Supplemental data

Supplemental data

Supplemental data

Supplemental data

Supplemental data

## Abbreviations Used

CDC: Centers for Disease Control and Prevention
CI: confidence interval
ICD-9: International Classification of Diseases, 9th edition
NIS: National Inpatient Sample
NYC: New York City
SD: standard deviation
SMM: severe maternal morbidity
