# Supplementary material for: Costs of Severe Maternal Morbidity in U.S. Commercially Insured and Medicaid Populations: An Updated Analysis
Source: Womens Health Rep (New Rochelle). 2021 Sep 27;2(1):443–51. doi: 10.1089/whr.2021.0026 (PMC8524749; doi:10.1089/whr.2021.0026)
Supplement: Supplemental data [file Supp_TableS1.docx]

**eTable 1. Codes for inclusion and exclusion in the study population**

|  | ICD-10 Diagnostic | ICD-10 Procedural | CPT | DRG |
| --- | --- | --- | --- | --- |
| Inclusion | Z37, O80, O300 | 10D07Z3, 0W8NXZZ with: 10D07Z4, 10D07Z5,10S07ZZ, 10D07Z6, 10D07Z8, 10D07Z7, 10E0XZZ, 0UL50ZZ, 0UL53ZZ, 0UL54ZZ, 0UL60ZZ, 0UL63ZZ, 0UL64ZZ, 0UL70ZZ, 0UL73ZZ, 0UL74ZZ, 10D00Z0, 10D00Z1, 10D00Z2 | 59409, 59612, 59514, 59620 | 767, 768, 774, 775, 765, 766 |
| Exclusion | O01, O02, O00, O03, O04, O07, O08, Z37.7 |  | 59840, 59841, 59850-59852, 59855-59857 | 770, 779, 777 |

CPT, current procedural terminology; DRG, diagnosis related group; ICD-10, International Classification of Diseases, Tenth Revision
