# Supplementary material for: Costs of Severe Maternal Morbidity in U.S. Commercially Insured and Medicaid Populations: An Updated Analysis
Source: Womens Health Rep (New Rochelle). 2021 Sep 27;2(1):443–51. doi: 10.1089/whr.2021.0026 (PMC8524749; doi:10.1089/whr.2021.0026)
Supplement: Supplemental data [file Supp_TableS2.docx]

### **eTable 2. SMM indicators and corresponding ICD-9 and short ICD-10 codes**

| Severe maternal morbidity indicator | DX/PR | ICD-9 | ICD-10 short |
| --- | --- | --- | --- |
| Acute myocardial infarction | DX | 410.xx | I21.xx, I22.x |
| Aneurysm ^a^ | DX | 441.xx | I71.xx, I79.0 ^b^ |
| Acute renal failure | DX | 584.5, 584.6, 584.7, 584.8, 584.9, 669.3x | N17.x, O90.4 |
| Adult respiratory distress syndrome | DX | 518.5x, 518.81 518.82 518.84, 799.1 | J80, J95.1, J95.2, J95.3, J95.82x, J96.0x, J96.2x R09.2 |
| Amniotic fluid embolism | DX | 673.1x | O88.1x |
| Cardiac arrest/ventricular fibrillation ^a^ | DX | 427.41, 427.42 ^c^, 427.5 | I46.x, I49.0x |
| Conversion of cardiac rhythm | PR | 99.6x | 5A2204Z, 5A12012 |
| Disseminated intravascular coagulation | DX | 286.6, 286.9, 666.3x | D65, D68.8, D68.9, O72.3 |
| Eclampsia | DX | 642.6x | O15. X |
| Heart failure/arrest during surgery or procedure | DX | 997.1 | I97.12x, I97.13x, I97.710, I97.711 |
| Puerperal cerebrovascular disorders | DX | 430.xx, 431.xx, 432.xx, 433.xx, 434.xx, 436xx, 437.xx, 671.5x, 674.0x, 997.02 | I60.xx- I68.xx, O22.51, O22.52, O22.53, I97.81x, I97.82x, O87.3 ^d^ |
| Pulmonary edema/ acute heart failure | DX | 518.4, 428.1, 428.0, 428.21, 428.23, 428.31, 428.33, 428.41, 428.43 | J81.0, I50.1, I50.20, I50.21, I50.23, I50.30, I50.31, I50.33, I50.40, I50.41, I50.43, I50.9 |
| Severe anesthesia complications | DX | 668.0x, 668.1x, 668.2x | O74.0, O74.1, O74.2, O74.3, O89.0x, O89.1, O89.2 |
| Sepsis | DX | 038.xx, 995.91, 995.92, 670.2x ^e^ | O85, O86.04, T80.211A, T81.4XXA, T81.44xx, or R65.20 or A40.x, A41.x, A32.7 |
| Shock | DX | 669.1x, 785.5x, 995.0, 995.4, 998.0x | O75.1, R57.x, R65.21, T78.2XXA, T88.2 XXA, T88.6 XXA, T81.10XA, T81.11XA, T81.19XA |
| Sickle cell disease with crisis | DX | 282.42, 282.62, 282.64, 282.69 | D57.0x, D57.21x, D57.41x, D57.81x |
| Air and thrombotic embolism | DX | 415.1x, 673.0x, 673.2x, 673.3x, 673.8x | I26.x, O88.0x, O88.2x, O88.3x, O88.8x |
| Blood products transfusion | PR | 99.0x | 30233 Peripheral vein, percutaneous (7th digit: x=1: non-autologous), 30240 Central Vein, open (7th digit: x=1: non-autologous), 30243 Central Vein, percutaneous (7th digit: x=1: non-autologous x=0: autologous)  +  Hx (whole blood)/ Kx (frozen plasma)/ Lx (fresh Plasma)/ Mx (plasma cryoprecipitate)/ Nx (red blood cells)/ Px (frozen Red cells)/ Rx (platelets)/ Tx (fibrinogen) Blood |
|  |  |  | Complete list of blood product transfusion codes:  ‘30233H1′, ’30233L1’, ‘30233K1’, ‘30233M1’, ‘30233N1′, ’30233P1′,’30233R1′, ’30233T1’, ‘30233H0′,’30233L0’, ‘30233K0’, ‘30233M0’, ‘30233N0′, ’30233P0′, ’30233R0′, ’30233T0’, ‘30230H1′, ’30230L1’, ‘30230K1’, ‘30230M1’, ‘30230N1′,’30230P1′,’30230R1′,’30230T1’,‘30230H0′, ’30230L0’, ‘30230K0’, ‘30230M0’, ‘30230N0′, ’30230P0′, ’30230R0′, ’30230T0’,‘30240H1′,’30240L1’, ‘30240K1’, ‘30240M1’, ‘30240N1′,’30240P1′,’30240R1′,’30240T1’,  ‘30240H0′,’30240L0’, ‘30240K0’, ‘30240M0’, ‘30240N0′, ’30240P0′, ’30240R0′, ’30240T0’, ‘30243H1′, ’30243L1’, ‘30243K1’, ‘30243M1’, ‘30243N1′, ’30243P1′, ’30243R1′, ’30243T1’, ‘30243H0′, ’30243L0’, ‘30243K0’, ‘30243M0’, ‘30243N0′, ’30243P0′, ’30243R0′, ’30243T0’, ‘30250H1′, ’30250L1’, ‘30250K1’, ‘30250M1’, ‘30250N1′, ’30250P1′, ’30250R1′, ’30250T1’, ‘30250H0′, ’30250L0’, ‘30250K0’, ‘30250M0’, ‘30250N0′, ’30250P0′, ’30250R0′, ’30250T0’,  ‘30253H1′, ’30253L1’, ‘30253K1’, ‘30253M1’, ‘30253N1′, ’30253P1′, ’30253R1′, ’30253T1’, ‘30253H0′, ’30253L0’, ‘30253K0’, ‘30253M0’, ‘30253N0′, ’30253P0′, ’30253R0′, ’30253T0’, ‘30260H1′, ’30260L1’, ‘30260K1’, ‘30260M1’, ‘30260N1′, ’30260P1′, ’30260R1′, ’30260T1’, ‘30260H0′, ’30260L0’, ‘30260K0’, ‘30260M0’, ‘30260N0′, ’30260P0′, ’30260R0′, ’30260T0’, ‘30263H1′, ’30263L1’, ‘30263K1’, ‘30263M1’, ‘30263N1′, ’30263P1′, ’30263R1′, ’30263T1’,  ‘30263H0′, ’30263L0’, ‘30263K0’, ‘30263M0’, ‘30263N0′, ’30263P0′, ’30263R0′, ’30263T0’ |
| Hysterectomy | PR | 68.3x-68.9x | 0UT90ZZ, 0UT94ZZ, 0UT97ZZ, 0UT98ZZ, 0UT9FZZ |
| Temporary tracheostomy ^a^ | PR | 31.1 | 0B110Z, 0B110F, 0B113, 0B114 |
| Ventilation | PR | 93.90, 96.01, 96.02, 96.03, 96.05 | 5A1935Z, 5A1945Z, 5A1955Z |

DX, diagnostic; ICD-10, International Classification of Diseases, Tenth Revision; PR, procedural; SMM, severe maternal morbidity

^a^ Due to rare prevalences, the following indicators are combined: aneurysm, cardiac arrest/ventricular fibrillation, and temporary tracheostomy.

^b^ No I71.7 code exists, so ICD-10 list encompasses all possible I71 codes.

^c^ Ventricular flutter.

^d^ 162.9 included, but should not be captured if this is not a valid code.

^e^ Code 670.2x used after October 1, 2009.

Adapted from: the CDC.^13^
