# Supplementary material for: Costs of Severe Maternal Morbidity in U.S. Commercially Insured and Medicaid Populations: An Updated Analysis
Source: Womens Health Rep (New Rochelle). 2021 Sep 27;2(1):443–51. doi: 10.1089/whr.2021.0026 (PMC8524749; doi:10.1089/whr.2021.0026)
Supplement: Supplemental data [file Supp_TableS3.docx]

### **eTable 3. Corresponding ICD-10 codes for preexisting comorbidities and obstetric-related complications**

| Description | ICD-10 |
| --- | --- |
| Preexisting comorbidities |  |
| Nonhereditary nonhemolytic anemia | D50.0, D51.0, D64.9 |
| Hereditary hemolytic anemia | D58.9 |
| Clotting disorders | D68.0, D69.6 |
| Tuberculosis | A15-A19 |
| Human immunodeficiency virus | B20 |
| Diabetes in pregnancy | O24 |
| Thyroid disorders | E00-E07 |
| Gall bladder disease | K80-K87 |
| Renal disease | N00-N07 |
| Liver disorders | K70-K77 |
| Asthma | J45 |
| Neurological conditions | G51.0 |
| Cardiovascular condition | I06-I09, I20, I34, I72, |
| Other chronic disease | K50, K51, M32, M06, P00.3, P00.89 |
| Mental health conditions | F30-39, F43 |
| Obesity | E66 |
| Chronic hypertension | I87.3 |
| Obstetric-related complications |  |
| Abnormal glucose tolerance | O99.814 |
| Amniotic sac | O41 |
| Cervical incompetence | O34.3 |
| Structural abnormality (uterus/cervix/vagina/vulva) | O65.5 |
| Excess vomiting | O21 |
| Placenta previa without hemorrhage | O44.0, O44.2 |
| Gestational hypertension/preeclampsia | O11.19 |
| Antepartum hemorrhage including placenta previa with hemorrhage | O44.3 |
| Postpartum hemorrhage | O72 |
| Pelvic and perineal trauma | O70-O71 |
| Uterine rupture | O71.1 |
| Obstetric infection | O86.0 |
| Urinary tract infection | O86.20 |
| Pneumonia | J15 |
| Appendicitis | K35 |
| Infections not classified elsewhere | B01.11, B15.0, J11.00 |
| Breast disorders | N61.0-N61.1, N64.0 |
| Complications of anesthesia | G97.1 |

ICD-10, International Classification of Diseases

Preexisting comorbidities and obstetric-related complications were identified from the literature.^21-23^
