# Supplementary material for: Costs of Severe Maternal Morbidity in U.S. Commercially Insured and Medicaid Populations: An Updated Analysis
Source: Womens Health Rep (New Rochelle). 2021 Sep 27;2(1):443–51. doi: 10.1089/whr.2021.0026 (PMC8524749; doi:10.1089/whr.2021.0026)
Supplement: Supplemental data [file Supp_TableS4.docx]

### **eTable 4. Median (IQR) costs without and with severe maternal morbidity in the commercial population, by maternal characteristics ^A^**

|  | Total | | |  | Prenatal | | |  | Delivery | | |  | Post-delivery | | |
| --- | --- | --- | --- | --- | --- | --- | --- | --- | --- | --- | --- | --- | --- | --- | --- |
|  | No SMM | SMM | Difference |  | No SMM | SMM | Difference |  | No SMM | SMM | Difference |  | No SMM | SMM | Difference |
| All patients | 19,078±12,518 | 32,120±31,910 | 13,042† |  | 3,746±4,923 | 7,252±14,699 | 3,506† |  | 12,071±6,429 | 15,880±11,902 | 3,809† |  | 1,150±2,942 | 2,915±9,850 | 1,765† |
| Age |  |  |  |  |  |  |  |  |  |  |  |  |  |  |  |
| 14-18 | 18,155±12,093 | 24,050±29,681 | 5,895 |  | 4,048±5,859 | 594±1,711 | -3,454† |  | 10,723±5,140 | 21,627±25,928 | 10,904† |  | 1,750±3,998 | 4,337±8,224 | 2,587 |
| 19-24 | 18,230±12,650 | 26,360±26,466 | 8,130† |  | 3,836±5,319 | 5,331±14,858 | 1,495† |  | 10,840±5,628 | 14,246±10,590 | 3,406† |  | 1,354±3,848 | 2,351±8,359 | 997† |
| 25-30 | 17,298±10,615 | 28,016±28,708 | 10,718† |  | 3,151±4,183 | 5,783±13,360 | 2,632† |  | 11,333±5,757 | 14,350±10,238 | 3,017† |  | 967±2,422 | 1,931±8,068 | 964† |
| 31-35 | 18,746±11,746 | 30,987±32,061 | 12,241† |  | 3,379±4,302 | 6,491±13,228 | 3,112† |  | 12,232±6,420 | 15,505±12,232 | 3,273† |  | 1,128±2,740 | 2,457±9,344 | 1,329† |
| 36-40 | 21,432±13,766 | 37,196±32,250 | 15,764† |  | 4,693±5,646 | 9,672±16,151 | 4,979† |  | 13,040±7,025 | 17,039±14,330 | 3,999† |  | 1,297±3,343 | 4,797±11,271 | 3,500† |
| 41-45 | 24,562±16,975 | 41,661±43,771 | 17,099† |  | 6,349±7,251 | 11,728±15,732 | 5,379† |  | 13,935±7,769 | 19,770±19,416 | 5,835† |  | 1,543±4,196 | 5,298±10,347 | 3,755† |
| >45 | 27,519±21,346 | 50,941±31,607 | 23,422† |  | 7,788±9,008 | 11,646±19,721 | 3,858 |  | 14,446±9,889 | 19,952±14,221 | 5,506† |  | 2,287±5,540 | 7,342±20,255 | 5,055† |
| Delivery method |  |  |  |  |  |  |  |  |  |  |  |  |  |  |  |
| Cesarean | 22,757±14,029 | 34,008±31,742 | 11,251† |  | 4,377±5,853 | 7,262±13,740 | 2,885† |  | 14,888±6,965 | 18,008±11,879 | 3,120† |  | 1,164±3,145 | 2,590±8,447 | 1,426† |
| Vaginal | 17,352±11,028 | 29,045±32,262 | 11,693† |  | 3,482±4,475 | 6,995±15,320 | 3,513† |  | 10,932±5,312 | 13,088±10,680 | 2,156† |  | 1,145±2,859 | 3,394±10,676 | 2,249† |
| Gestation type |  |  |  |  |  |  |  |  |  |  |  |  |  |  |  |
| Singleton | 18,678±11,854 | 30,583±29,518 | 11,905† |  | 3,570±4,579 | 6,464±13,528 | 2,894† |  | 11,942±6,232 | 15,469±11,489 | 3,527† |  | 1,132±2,858 | 2,701±9,316 | 1,569† |
| Multifetal | 29,296±22,264 | 54,360±48,638 | 25,064† |  | 9,266±12,190 | 19,475±32,458 | 10,209† |  | 14,771±8,852 | 17,773±11,984 | 3,002† |  | 1,433±4,315 | 5,607±16,278 | 4,174† |
| Region |  |  |  |  |  |  |  |  |  |  |  |  |  |  |  |
| Midwest | 17,596±10,021 | 30,189±31,802 | 12,593† |  | 3,471±4,254 | 7,887±15,770 | 4,416† |  | 1,1171±4,857 | 14,464±9,720 | 3,293† |  | 1,177±3,052 | 3,552±10,777 | 2,375† |
| North East | 24,619±14,811 | 43,436±41,076 | 18,817† |  | 5,208±6,109 | 10,437±17,432 | 5,229† |  | 15,605±8,310 | 22,080±15,822 | 6,475† |  | 1,412±3,389 | 4,415±12,818 | 3,003† |
| South East | 17,460±10,492 | 32,971±32,375 | 15,511† |  | 3,283±4,399 | 7,715±15,545 | 4,432† |  | 11,164±5,416 | 14,893±10,501 | 3,729† |  | 1,071±2,761 | 3,702±10,654 | 2,631† |
| West | 21,083±14,744 | 26,479±24,998 | 5,396† |  | 3,940±5,482 | 5,216±7,999 | 1,276† |  | 13,638±8,148 | 15,647±12,207 | 2,009 |  | 1,082±2,733 | 1,651±6,510 | 569 |

^A^ Data from MarketScan® Commercial Claims and Encounters database. Difference of medians tested using Wilcoxon-Rank Sum Test (2 level) or Kruskal-Wallis Test (3 or more levels) of median costs.

†P<0.001.
