# Supplementary material for: Costs of Severe Maternal Morbidity in U.S. Commercially Insured and Medicaid Populations: An Updated Analysis
Source: Womens Health Rep (New Rochelle). 2021 Sep 27;2(1):443–51. doi: 10.1089/whr.2021.0026 (PMC8524749; doi:10.1089/whr.2021.0026)
Supplement: Supplemental data [file Supp_TableS5.docx]

**eTable 5. Median (IQR) costs without and with severe maternal morbidity in the Medicaid population, by maternal characteristics ^A^**

|  | Total | | |  | Prenatal | | |  | Delivery | | |  | Post-delivery | | |
| --- | --- | --- | --- | --- | --- | --- | --- | --- | --- | --- | --- | --- | --- | --- | --- |
|  | No SMM | SMM | Difference |  | No SMM | SMM | Difference |  | No SMM | SMM | Difference |  | No SMM | SMM | Difference |
| All patients | 7,786±4,900 | 16,163±19,655 | 8,377† |  | 2,332±2,431 | 5,527±8,398 | 3,195† |  | 3,606±1,423 | 5,260±3,239 | 1,654† |  | 1,271±2,813 | 3,279±7,672 | 2,008† |
| Age |  |  |  |  |  |  |  |  |  |  |  |  |  |  |  |
| 14-18 | 8,162±4,489 | 15,820±11,944 | 7,658† |  | 2,369±2,191 | 3,325±2,688 | 956 |  | 3,512±1,108 | 5,523±3,280 | 2,011† |  | 1,741±2,552 | 2,231±6,411 | 490 |
| 19-24 | 7,818±4,713 | 14,756±17,726 | 6,938† |  | 2,388±2,356 | 4,602±8,837 | 2,214† |  | 3,551±1,320 | 5,087±2,528 | 1,536† |  | 1,403±2,681 | 3,837±6,857 | 2,434† |
| 25-30 | 7,703±4,855 | 17,363±20,012 | 9,660† |  | 2,296±2,464 | 5,818±8,167 | 3,522† |  | 3,606±1,435 | 5,304±3,293 | 1,698† |  | 1,187±2,851 | 3,838±10,445 | 2,651† |
| 31-35 | 7,749±5,248 | 15,830±20,105 | 8,081† |  | 2,275±2,558 | 6,263±7,419 | 3,988† |  | 3,677±1,427 | 5,532±3,732 | 1,855† |  | 1,095±2,936 | 2,469±7,505 | 1,374† |
| 36-40 | 8,009±5,406 | 15,676±25,679 | 7,667† |  | 2,291±2,600 | 5,325±10,349 | 3,034† |  | 3,839±1,625 | 5,632±3,208 | 1,793† |  | 1,079±2,939 | 3,569±12,578 | 2,490† |
| 41-45 | 7,591±5,811 | 18,587±17,948 | 10,996† |  | 2,343±2,545 | 8,510±7,220 | 6,167† |  | 3,826±1,510 | 5,505±4,253 | 1,679† |  | 821±2,846 | 6,204±10,289 | 5,383† |
| >45 | 9,282±6,353 | 0±0 | -9,282 |  | 2,186±3,668 | 0±0 | -2,186 |  | 4,208±1,614 | 0±0 | -4,208 |  | 1,233±3,901 | 0±0 | -1,233 |
| Delivery method |  |  |  |  |  |  |  |  |  |  |  |  |  |  |  |
| Cesarean | 9,037±5,549 | 15,874±20,392 | 6,837† |  | 2,607±2,764 | 4,496±6,327 | 1,889† |  | 4,455±1,193 | 5,854±4,066 | 1,399† |  | 1,280±3,028 | 2,297±7,147 | 1,017† |
| Vaginal | 7,343±4,521 | 16,888±18,970 | 9,545† |  | 2,240±2,328 | 6,793±9,837 | 4,553† |  | 3,396±968 | 4,701±2,812 | 1,305† |  | 1,267±2,743 | 4,075±7,741 | 2,808† |
| Gestation type |  |  |  |  |  |  |  |  |  |  |  |  |  |  |  |
| Singleton | 7,702±4,729 | 16,163±18,253 | 8,461† |  | 2,290±2,356 | 6,241±8,934 | 3,951† |  | 3,606±1,389 | 5,107±2,622 | 1,501† |  | 1,256±2,772 | 3,940±7,271 | 2,684† |
| Multifetal | 10,290±6,196 | 13,379±28,074 | 3,089† |  | 3,500±3,690 | 4,351±14,594 | 851 |  | 4,415±1,934 | 6,353±3,738 | 1,938† |  | 1,478±3,095 | 2,131±3,678 | 653 |
| Race/ Ethnicity |  |  |  |  |  |  |  |  |  |  |  |  |  |  |  |
| Black | 7,975±4,587 | 15,955±1,9437 | 7,980† |  | 2,392±2,388 | 5,818±8,812 | 3,426† |  | 3,656±1,382 | 5,214±2,392 | 1,558† |  | 1,353±2,619 | 3,279±7,095 | 1,926† |
| Hispanic | 7,020±3,645 | 16,008±1,5189 | 8,988† |  | 1,938±1,826 | 4,312±8,698 | 2,374† |  | 3,579±1,359 | 5,023±2,208 | 1,444† |  | 1,004±2,053 | 4,623±8,098 | 3,619† |
| Other | 7,052±4,537 | 18,350±1,0078 | 11,298† |  | 1,867±2,181 | 3,831±6,614 | 1,964† |  | 3,606±1,487 | 7,255±4,315 | 3,649† |  | 1,008±2,252 | 3,300±5,007 | 2,292 |
| White | 9,423±8,186 | 34,138±3,4163 | 24,715† |  | 2,412±3,741 | 13,081±18,368 | 10,669† |  | 4,963±1,932 | 7,784±4,064 | 2,821† |  | 1,289±4,059 | 15,484±20,719 | 14,195† |

^A^ Data from MarketScan® Medicaid database. Difference of medians tested using Wilcoxon-Rank Sum Test (2 level) or Kruskal-Wallis Test (3 or more levels) of median costs.

†P<0.001.
